# Experimental Study on the Mechanical Properties of Rice Husk-Reinforced Eco-Friendly Mine Backfill Material

**DOI:** 10.3390/ma19143121

**Published:** 2026-07-21

**Authors:** Jinxing Lyu, Bao Song, Yiquan Lin, Wen Ma, Songxiang Liu

**Affiliations:** 1 School of Geology and Mining Engineering, Xinjiang University, Urumqi 830046, China; 107552404741@stu.xju.edu.cn (B.S.); linquan0821@163.com (Y.L.); 107552504754@stu.xju.edu.cn (W.M.); lsx8856528@stu.xju.edu.cn (S.L.); 2 School of Mines, China University of Mining and Technology, Xuzhou 221116, China

**Keywords:** rice husk, desert sand, coal gangue, cemented backfill, damage evolution

## Abstract

This study developed an eco-friendly mine backfill material using coal gangue, fly ash, desert sand, and natural rice husk. The mechanical properties and damage evolution characteristics were investigated through uniaxial compression, Brazilian splitting, acoustic emission (AE), digital image correlation (DIC), and scanning electron microscopy (SEM) tests. Polyvinyl alcohol (PVA) fibers provided the highest reinforcement efficiency, followed by polypropylene (PP) fibers. At a natural rice husk dosage of 5%, the backfill reached a compressive strength of 5.6 MPa and a splitting tensile strength of 0.376 MPa, representing an 83.4% increase in tensile strength compared with the fiber-free group and approaching the performance of the PP fiber group. Increasing the cement replacement ratio from 50% to 70% reduced strength, whereas longer curing improved strength development. The coupled AE–DIC observations indicate that natural rice husk incorporation delayed crack localization and changed the failure process from sudden brittle cracking to more progressive damage. SEM observations show that natural rice husk formed local contact and partial embedding with the cementitious matrix, with hydration products around the rice husk and visible pull-out voids. Overall, natural rice husk can be used as a low-cost and sustainable reinforcing material for coal gangue–desert sand–fly ash-based mine backfill.

## 1. Introduction

With the continued advancement of high-intensity coal mining and green mine construction, the large-scale generation and stockpiling of coal-based solid wastes, such as coal gangue and fly ash, have become prominent environmental and engineering concerns [1]. Among these wastes, coal gangue is a typical bulk industrial solid waste generated during coal mining and processing. Its cumulative historical stockpile has exceeded 7 billion tons, while its current comprehensive utilization rate is only about 60%, and the annual newly generated amount remains as high as 600–800 million tons. Coal gangue generally accounts for 10–25% of raw coal production and is one of the largest industrial solid wastes in China [2]. The extensive accumulation of coal-based solid wastes not only occupies large areas of land but may also cause dust pollution, leachate migration, groundwater contamination, and long-term ecological risks during weathering, rainfall leaching, and open-air storage. Cemented backfill mining can consume solid wastes such as coal gangue, fly ash, and tailings while filling underground goaf areas, thereby controlling surface subsidence and surrounding rock deformation. Therefore, it represents an important technical approach for green mine construction and sustainable underground mining [3,4].

In recent years, the resource utilization of coal-based solid wastes, such as coal gangue and fly ash, in cemented backfill materials has received increasing attention. Existing studies have mainly focused on mechanical properties, microstructural evolution, service-environment effects, and engineering flowability. In terms of mechanical properties and microstructure, coal gangue and fly ash can be used as aggregates or supplementary cementitious components in backfill materials, and their strength development is affected by factors such as curing temperature, fly ash content, and the formation of hydration products [5,6]. Under complex service environments and special curing conditions, coupled hydraulic pressure–chloride erosion and low-temperature curing can alter the pore structure, damage evolution, and load-bearing performance of backfill materials, thereby influencing their long-term stability [7,8]. In addition, cemented backfill materials must satisfy the flow and transport requirements of stope backfilling, and their yield stress and lubrication flow behavior directly affect their engineering applicability [9]. For composite cementitious systems containing multiple solid wastes, the synergistic interactions among different waste components can further influence the hydration mechanism and the optimization of mechanical properties [10]. Overall, previous studies have demonstrated the promising potential of coal gangue and fly ash for resource utilization in mine backfill materials. However, coal-based solid-waste cemented backfill still suffers from pronounced brittle failure, insufficient post-peak load-bearing capacity, and limited crack resistance. Therefore, improving the toughness and crack resistance of backfill materials while maintaining a high solid-waste utilization rate and good engineering adaptability remains a key issue that requires further investigation.

To address the pronounced brittleness, limited crack resistance, and insufficient post-peak deformation capacity of coal-based solid-waste cemented backfill materials, fiber modification has gradually become an important approach for improving their toughness and damage tolerance. In terms of mechanical reinforcement, Han et al. [11] investigated the effects of ordinary steel fibers and micro steel fibers on the fracture properties of spontaneous-combustion coal gangue aggregate concrete. Their results showed that steel fibers could improve the brittle failure characteristics of the material, with micro steel fibers producing a more pronounced enhancement in fracture toughness and fracture energy. Zhang et al. [12] further studied the effects of polypropylene fiber content and curing age on the flexural performance of coral aggregate concrete, and reported that an appropriate amount of polypropylene fiber could form a randomly distributed three-dimensional network within the matrix, thereby improving the flexural strength and deformation capacity of the material. For damage control in backfill materials, Wang et al. [13] found that an appropriate basalt fiber content could improve the pore structure of cemented tailings backfill, increase its peak stress and peak strain, and delay crack coalescence. Zhao et al. [14] investigated the failure mechanism of cement–fiber–tailings matrix composites by combining digital image correlation and acoustic emission techniques, and showed that fiber incorporation helped improve the damage evolution process and failure characteristics of the backfill. Li et al. [15] reported that basalt fibers and polyvinyl alcohol fibers could enhance the load-bearing capacity and crack resistance of consolidated bodies through their interaction with the solid-waste slurry matrix. In addition, from the perspective of fresh slurry transport performance, Yu et al. [16] showed that the type and content of virgin and recycled polymer fibers changed the slump, apparent viscosity, and rheological properties of cemented paste backfill to varying degrees. This indicates that fiber-reinforced backfill materials should be evaluated not only in terms of hardened mechanical properties, but also in terms of slurry flowability and field transport adaptability. Overall, conventional fibers such as steel fibers, polypropylene fibers, basalt fibers, and polyvinyl alcohol fibers can effectively improve the toughness and crack resistance of cement-based materials and backfill materials. In contrast, plant-based natural fibers have attracted increasing attention because of their renewability, low density, and potential environmental advantages [17,18]. Therefore, the development of low-cost, renewable, and environmentally friendly natural reinforcing materials is of great significance for constructing green and low-carbon mine backfill materials.

Rice husk is a typical lignocellulosic agricultural waste with wide availability, low density, a rough outer surface, and certain toughness. These characteristics give it potential as a natural reinforcing phase in cement-based composites. Previous studies on plant-fiber- and natural-fiber-reinforced cement-based materials have also shown that such materials can contribute to crack control, toughness improvement, and deformation capacity enhancement [19,20]. Meanwhile, the interfacial stability of lignocellulosic materials in cementitious environments and their interaction with the matrix are important factors affecting the reinforcing effect. Related studies have also demonstrated that natural fibers and cellulose-based materials can be used to improve the performance of solid-waste backfill materials and civil engineering composites [21,22,23]. However, existing studies on the resource utilization of rice husk have mainly focused on treated rice-husk-based materials. For example, rice husk ash is usually obtained by high-temperature calcination of rice husk, and it mainly acts as an active mineral admixture or fine filler in cementitious systems. Its contribution to material performance is generally associated with pozzolanic reactions, micro-filling effects, and the regulation of hydration products [24]. In addition, some studies have used rice husk biomass or rice husk biochar in soil stabilization, lightweight fillers, and subgrade materials, with emphasis on their filling, stabilization, or lightweight functions [25,26]. Compared with rice husk ash, untreated natural rice husk is not subjected to calcination and therefore retains its lignocellulosic structure, rough surface, and irregular morphology to some extent. Its role in cemented backfill materials may not depend primarily on pozzolanic reactions or micro-filling effects, but may be more closely related to its physical influence on matrix interfaces and crack development. In addition, the morphology and size distribution of natural rice husk differ from those of common linear natural fibers. Its dispersion state in the slurry, interfacial bonding with the matrix, and dosage may all affect the material performance. An appropriate amount of natural rice husk is expected to improve crack propagation behavior and deformation coordination of the backfill, whereas excessive incorporation may lead to increased porosity, non-uniform dispersion, or local interfacial weakening. Therefore, the role of natural rice husk in coal gangue–fly ash-based cemented backfill systems cannot be fully inferred from studies on rice husk ash or other natural fibers. Its reinforcing effect, suitable dosage, and interfacial interaction still require further investigation.

In addition to natural reinforcing materials, aggregate sources are also important factors affecting the environmental performance and engineering applicability of backfill materials. For arid and semi-arid mining regions such as Xinjiang, desert sand has the practical advantage of local availability. Using desert sand as a fine aggregate in backfill materials can reduce the consumption of natural river sand and decrease the demand for long-distance transportation to some extent [27]. Previous studies have shown that the incorporation or replacement ratio of desert sand can influence the mechanical properties, deformation and failure characteristics, and engineering applicability of cement-based materials, and its effect is closely related to the dosage and matrix composition [28,29]. However, most existing studies have focused on the application of desert sand in ordinary cement-based materials or concrete systems, while the mechanical behavior of composite backfill materials containing desert sand, natural rice husk, coal gangue, and fly ash remains insufficiently understood. Furthermore, under the same backfill material system, systematic comparisons between natural rice husk and conventional fibers, such as PP and PVA, in terms of strength, deformation characteristics, and failure process are still limited. Therefore, investigations combining macroscopic mechanical response, deformation and failure process, and micro-damage evolution are useful for evaluating the performance of reinforced backfill materials [30,31,32]. Such a combined approach is also necessary to clarify the applicability and potential role of natural rice husk in the composite backfill system investigated in this study.

To address these research gaps, this study prepared a natural rice husk-reinforced eco-friendly mine backfill material using coal gangue, fly ash, cement, desert sand, and natural rice husk as the main raw materials, with PP and PVA fiber groups designed for comparison. Compared with previous studies, this work further focuses on the application of natural rice husk in a composite backfill system composed of natural rice husk, desert sand, coal gangue, and fly ash, and compares its performance with those of conventional fiber-reinforced materials, including PP and PVA, under the same mixture proportions and curing conditions. Specifically, the effects of fiber type, natural rice husk content, cement replacement ratio, and curing age on the compressive strength, splitting tensile strength, and stress–strain behavior of the backfill material were systematically investigated. In addition, AE, DIC, and SEM tests were conducted to analyze the role of natural rice husk in reinforced backfill materials from the perspectives of macroscopic mechanical response, crack evolution process, and microstructural characteristics. This study provides a reference for the synergistic resource utilization of agricultural waste, coal-based solid waste, and desert sand in mine backfilling.

## 2. Experimental Section

### 2.1. Raw Materials

The primary raw materials used in this study included coal gangue, desert sand, cement, fly ash, natural rice husk, polypropylene (PP) fibers, polyvinyl alcohol (PVA) fibers, and tap water. Coal gangue was obtained from a mine in Tacheng, Xinjiang. It was crushed using a jaw crusher (Hebi Metallurgical Machinery Co., Ltd., Hebi, China), ground in a ball mill (Changsha Miqi Instrument Co., Ltd., Changsha, China), and sieved for subsequent use. The oxide composition of coal gangue was determined using an X-ray fluorescence (XRF) spectrometer (NCS Testing Technology Co., Ltd., Beijing, China), and the results are listed in Table 1. The coal gangue was mainly composed of SiO_2_ and Al_2_O_3_, with mass fractions of 57.68% and 26.97%, respectively. In addition, small amounts of Fe_2_O_3_, K_2_O, CaO, TiO_2_, SO_3_, MgO, Na_2_O, and P_2_O_5_ were also detected. Desert sand was collected from Fukang, Xinjiang, and sieved to be used as fine aggregate. Particle size distribution tests indicated that D10, D50, and D90 values were 103.4 μm, 199.5 μm, and 309.9 μm, respectively, with a volume mean particle size of 201.0 μm. The particle size distribution of the coal gangue aggregates is summarized in Table 2.

Cement used in this study was ordinary Portland cement (P.O 42.5; Xinjiang Tianshan Cement Co., Ltd., Urumqi, China). The chemical composition of the cement was determined by XRF analysis, and its mineralogical composition was identified using an X-ray diffractometer (CIQTEK Co., Ltd., Hefei, China). The results are summarized in Table 3 and Table 4. The cement was mainly composed of CaO and SiO_2_, with mass fractions of 56.60% and 24.70%, respectively, together with certain amounts of Al_2_O_3_ and Fe_2_O_3_. The main mineral phases were tricalcium silicate (C_3_S), dicalcium silicate (C_2_S), tricalcium aluminate (C_3_A), and tetracalcium aluminoferrite (C_4_AF), indicating a typical Portland cement system.

The fly ash used in this study was Class I fly ash produced by Gongyi Longze Water Purification Materials Co., Ltd. (Gongyi, China), and was used as a supplementary cementitious material to partially replace cement. Its main physical properties were provided by the manufacturer and are listed in Table 5. To further characterize the chemical and mineralogical features of the fly ash, XRF and XRD analyses were conducted using the same instruments described above. The corresponding results are presented in Table 6 and Table 7. The XRF results show that the fly ash was mainly composed of SiO_2_ and Al_2_O_3_, with mass fractions of 49.08% and 36.29%, respectively, together with smaller amounts of Fe_2_O_3_, CaO, K_2_O, TiO_2_, SO_3_, MgO, Na_2_O, and P_2_O_5_. The XRD results indicate that mullite and quartz were the main crystalline phases, accounting for 95.23% and 4.77%, respectively.

Natural rice husk was collected from agricultural waste in Baoji, Shaanxi, and prepared by washing, drying, and sieving for subsequent use. Both polypropylene (PP) fibers and polyvinyl alcohol (PVA) fibers were short-cut fibers produced by Shanghai Chenqi Chemical Technology Co., Ltd. (Shanghai, China). The main properties of these reinforcement materials are summarized in Table 8. Tap water was used as the mixing water.

### 2.2. Mix Design and Specimen Preparation

This study adopted a single-factor, multi-level comparative experimental design, with the slurry mass concentration fixed at 78% and the sand-to-binder ratio maintained at 2:1. First, under a cement replacement ratio of 50% and a curing age of 28 days, six groups were designed: a fiber-free group, PP-1%, PVA-1%, and rice husk groups with dosages of 1%, 5%, and 10%. These groups were used to evaluate the effects of different fiber types and rice husk dosages on the mechanical properties of the cemented backfill. On this basis, the group containing 5% rice husk was selected as the representative rice-husk-reinforced group. Additional test groups with cement replacement ratios of 60% and 70% were then prepared, and comparative tests were conducted at curing ages of 3, 7, and 28 days. In total, 10 test conditions were established, as detailed in Table 9.

For each test condition, three specimens were prepared for uniaxial compression tests and three specimens for Brazilian splitting tests, giving a total of 60 specimens. The specimens were named according to the format “reinforcing material and dosage–C cement replacement ratio–curing age”. In this notation, NF denotes the fiber-free group, while RH, PP, and PVA represent rice husk, polypropylene fiber, and polyvinyl alcohol fiber, respectively. The symbol C denotes the cement replacement ratio. For example, RH5-C50-28d refers to a specimen containing 5% rice husk, with a cement replacement ratio of 50% and a curing age of 28 days.

Prior to mixing, the polypropylene (PP) and polyvinyl alcohol (PVA) fibers were lightly shaken and pre-dispersed to reduce the risk of fiber agglomeration, while the natural rice husk was washed, dried, and sieved for subsequent use. According to the designed mix proportions, desert sand, coal gangue, cement, and fly ash were initially placed in a mixer (Changzhou Guohua Electric Appliance Co., Ltd., Changzhou, China) and dry-mixed at low speed for approximately 1 min to achieve preliminary uniformity between the aggregates and cementitious materials. Subsequently, rice husk, PP fibers, or PVA fibers were added and further mixed to ensure even dispersion of the reinforcement materials within the dry blend. Finally, the measured water was slowly incorporated, and mixing continued until a homogeneous paste was obtained.

**Figure 1:**
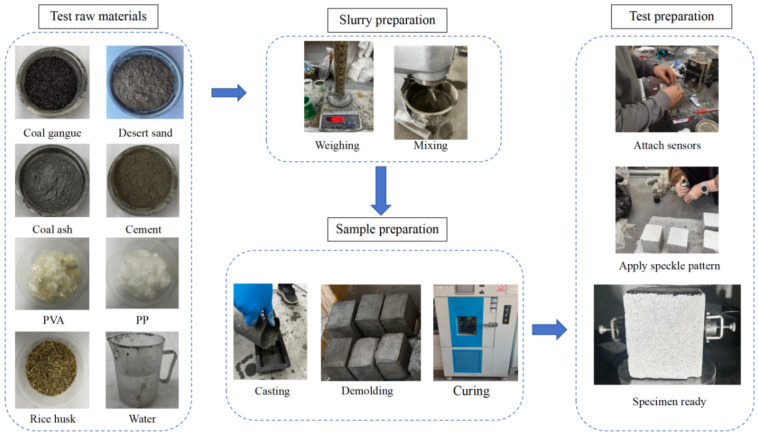
Raw materials and specimen preparation process.

The prepared mixture was poured into a three-part mold measuring 100 mm × 100 mm × 100 mm in two layers. After placing each layer, a vibration table (Wuxi Huaxi Instrument Co., Ltd., Wuxi, China) was used to compact the material for 15–30 s until the surface appeared glossy and free of noticeable air bubbles. Upon completion of mold filling, the surface was leveled and covered with plastic film to minimize early moisture loss. Specimens were left at room temperature for 24 h before demolding, and then cured in a controlled curing chamber (Shanghai Luda Experimental Instrument Co., Ltd., Shanghai, China) at 20 ± 2 °C with a relative humidity of 95%. Specimens were retrieved for testing at specified curing ages of 3, 7, and 28 days. The specimen preparation procedure is illustrated in Figure 1.

### 2.3. Test Methods

**Figure 2:**
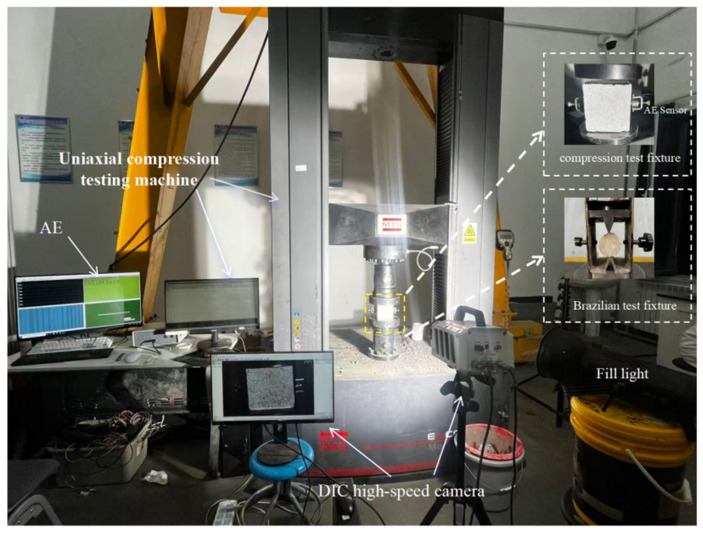
Testing and monitoring system.

Uniaxial compression tests were conducted on the backfill specimens using a WAW-1000 microcomputer-controlled electro-hydraulic servo universal testing machine (Hangzhou MTS Testing Equipment Co., Ltd., Hangzhou, China), while the splitting tensile tests were performed using a dedicated Brazilian test fixture supplied by the same manufacturer. To better understand the deformation and failure modes of the specimens, the failure process was monitored using an acoustic emission (AE) system (Beijing Soft Island Times Technology Co., Ltd., Beijing, China) combined with digital image correlation (DIC) techniques. Displacement-controlled testing was adopted to allow detailed observation of specimen failure, with a displacement rate set at 0.5 mm/min. AE sensors were attached to the specimen surfaces to capture acoustic emission signals generated during crack initiation and propagation, which were transmitted to the recording system. The actual test and monitoring system setup is shown in Figure 2.

(1) Acoustic Emission (AE) is a non-destructive testing (NDT) technique used to detect stress, deformation, or damage-induced acoustic signals in materials or structures. When cracks propagate, plastic deformation occurs, or other forms of energy are rapidly released within the material, ultrasonic waves or acoustic pulses are generated. These signals can be captured and analyzed by the AE system. Based on the propagation velocity of sound in the material, the specimens were initially classified and located according to their acoustic velocities. Two AE sensors were then positioned on the sides and rear of each specimen, forming three spatial planes to capture signals effectively.

(2) Digital Image Correlation (DIC) is a full-field, non-contact experimental technique based on optical measurement principles. DIC determines the two-dimensional or three-dimensional strain field, displacement field, and related mechanical parameters by comparing surface images of the specimen before and during loading. In this study, a high-speed camera (Qianyanlang, Hefei Fuhuang Junda High-Tech Information Technology Co., Ltd., Hefei, China) with a resolution of 2048 × 1536 pixels was placed in front of the concrete specimen, with side lighting to ensure uniform illumination. Images were captured at a rate of one frame per second, allowing real-time observation and recording of specimen morphology and crack propagation during failure.

## 3. Results and Discussion

### 3.1. Effects of Fiber Type and Rice Husk Content on Mechanical Properties

**Figure 3:**
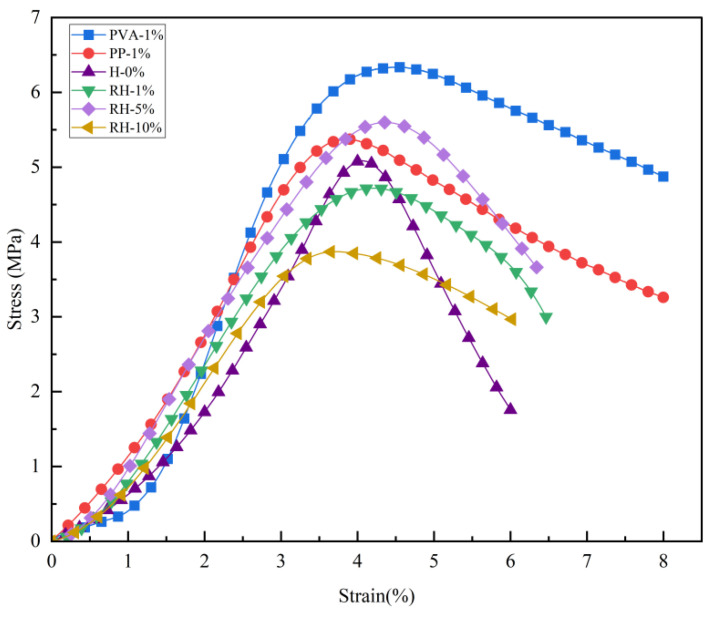
Typical stress-strain curves of backfill with different fiber types and rice husk contents.

**Figure 4:**
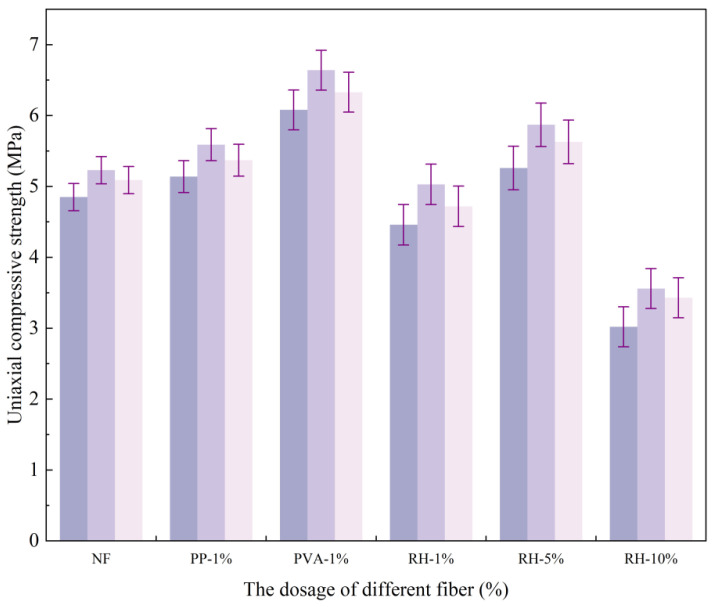
Compressive strength of backfill with different fiber types and rice husk contents.

**Figure 5:**
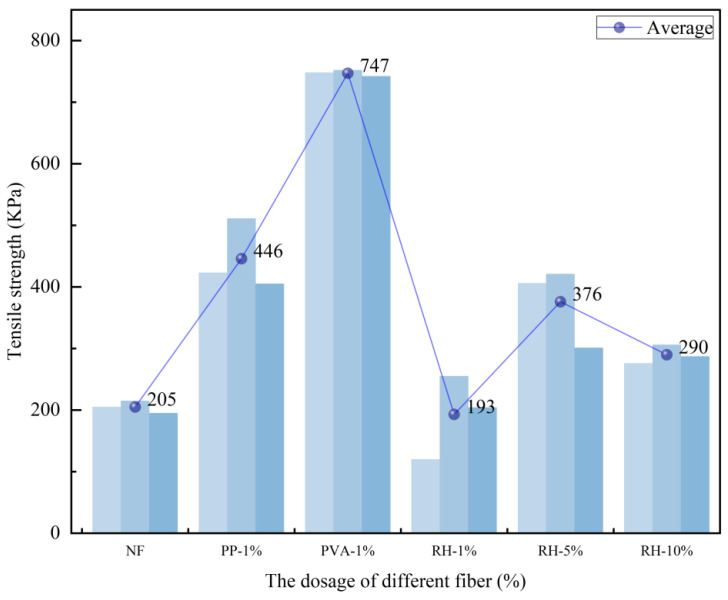
Tensile strength of backfill with different fiber types and rice husk contents. Note: All groups in Figure 3, Figure 4 and Figure 5 had a cement replacement ratio of 50% and a curing age of 28 d.

To investigate the effects of natural rice husk fibers and conventional synthetic fibers on the mechanical properties of cemented backfill, comparative tests were conducted under a slurry mass concentration of 78%, a sand-to-binder ratio of 2:1, a cement replacement ratio of 50%, and a curing age of 28 days. The effects of different fiber types and rice husk dosages on the mechanical properties of the backfill are shown in Figure 3, Figure 4 and Figure 5. As shown in Figure 3, the stress–strain curves of all specimens exhibit typical staged characteristics, including compaction, elastic deformation, plastic development, and post-peak failure. However, the fiber type and rice husk dosage have pronounced effects on the peak stress, post-peak load-bearing capacity, and overall curve shape.

As shown in Figure 3, Figure 4 and Figure 5, the fiber-free group H-0% exhibits a compressive strength of approximately 5.0 MPa and an average tensile strength of 0.205 MPa. Its stress–strain curve drops rapidly after the peak, indicating a marked reduction in post-peak load-bearing capacity and a typical brittle failure behavior. After the incorporation of synthetic fibers, the mechanical properties of the specimens are improved to varying degrees. Among them, the PVA-1% group shows the most significant enhancement, with a compressive strength of approximately 6.5 MPa and an average tensile strength of 0.747 MPa, corresponding to increases of approximately 30.0% and 264.4%, respectively, compared with the H-0% group. The PP-1% group also exhibits improved performance, with a compressive strength of approximately 5.4 MPa and an average tensile strength of 0.446 MPa, representing increases of approximately 8.0% and 117.6%, respectively, relative to the H-0% group. Combined with the stress–strain curves, the more gradual post-peak descending branches of the PVA-1% and PP-1% groups suggest that synthetic fibers may help delay rapid crack propagation and improve the post-peak deformation capacity and crack resistance of the backfill.

The RH-1% group shows a compressive strength of approximately 4.8 MPa and an average tensile strength of 0.193 MPa, which are close to those of the H-0% group. This indicates that a low rice husk dosage has a limited influence on the mechanical improvement of the backfill. When the rice husk dosage increases to 5%, the compressive strength of the RH-5% group increases to approximately 5.6 MPa, which is slightly higher than that of the PP-1% group. Its average tensile strength reaches 0.376 MPa, representing an increase of approximately 83.4% compared with the H-0% group and approaching that of the PP-1% group. Meanwhile, the post-peak decline of the stress–strain curve for the RH-5% group is more gradual than those of the RH-1% and RH-10% groups. This result suggests that an appropriate rice husk dosage may help improve the post-peak deformation behavior and crack resistance of the backfill, which is likely related to the interaction between rice husk and the cementitious matrix during crack development.

However, when the rice husk dosage is further increased to 10%, the compressive strength of the RH-10% group decreases to approximately 3.5 MPa, which is about 37.5% lower than that of the RH-5% group. Its tensile strength is 0.290 MPa, approximately 22.9% lower than that of the RH-5% group, suggesting that excessive rice husk content is unfavorable for strength development. This may be attributed to the fact that excessive rice husk can disturb the continuity and compactness of the cementitious matrix and increase the possibility of local defects or weak interfacial zones. Therefore, the reinforcing effect of rice husk appears to depend on a balance between its potential crack-resistance contribution and its influence on matrix integrity. Under the present experimental conditions, RH-5% provides a relatively favorable balance among compressive strength, tensile strength, and post-peak deformation behavior, and was therefore selected as the representative rice husk dosage for subsequent analysis.

### 3.2. Effects of Cement Replacement Ratio and Curing Age on Mechanical Properties

**Figure 6:**
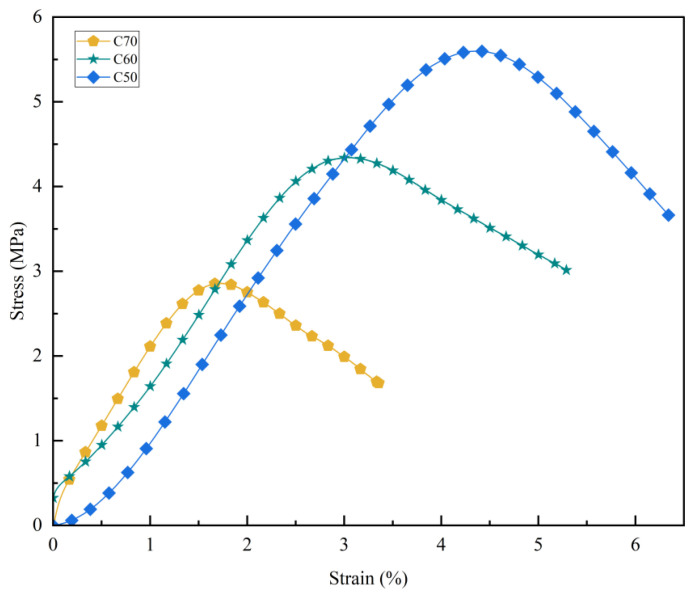
Typical stress-strain curves at different cement replacement ratios. Note: C50, C60 and C70 denote cement replacement ratios of 50%, 60% and 70%, respectively. All groups had 5% rice husk and a curing age of 28 d.

**Figure 7:**
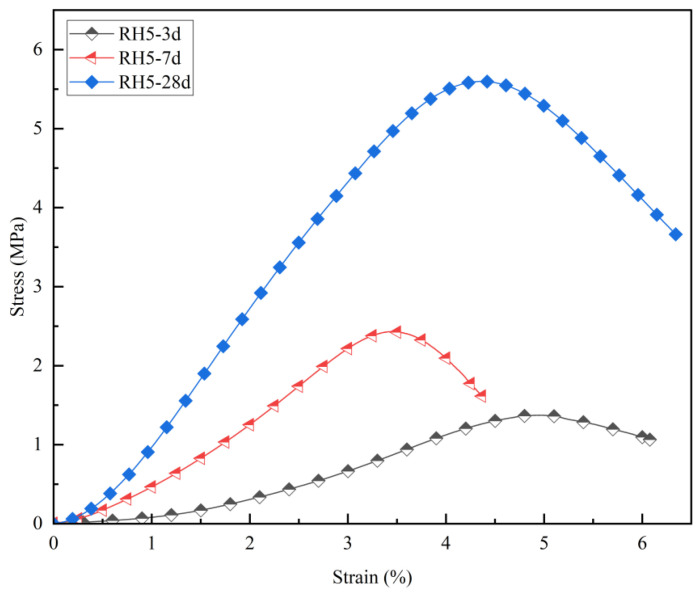
Typical stress-strain curves at different curing ages. Note: All groups in Figure 7 had 5% rice husk and a cement replacement ratio of 50%.

**Figure 8:**
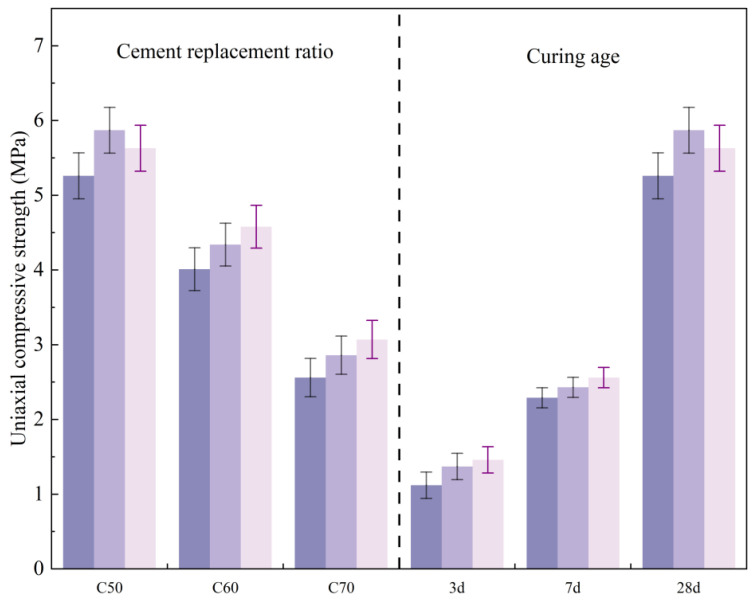
Uniaxial compressive strength under different cement replacement ratios and curing ages.

**Figure 9:**
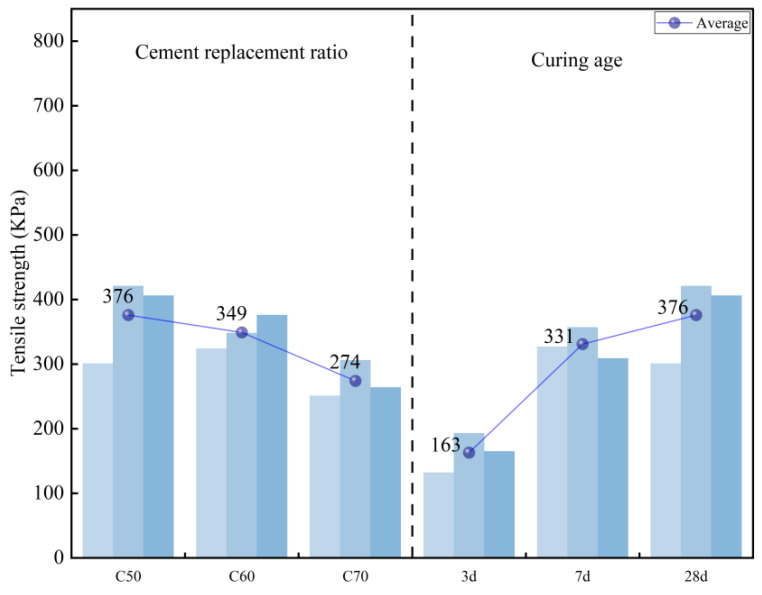
Tensile strength under different cement replacement ratios and curing ages. Note: In Figure 8 and Figure 9, the left-side groups had 5% rice husk and a curing age of 28 d, and the right-side groups had 5% rice husk and a cement replacement ratio of 50%.

To further investigate the effects of binder composition and curing age on the mechanical properties of the backfill at a rice husk dosage of 5%, the typical stress–strain curves, uniaxial compressive strength, and tensile strength of specimens with cement replacement ratios of 50%, 60%, and 70% and curing ages of 3, 7, and 28 days were examined. The results are presented in Figure 6, Figure 7, Figure 8 and Figure 9.

As the cement replacement ratio increased from 50% to 70%, the peak stress of the stress–strain curves gradually decreased, and both the compressive and tensile strengths showed a declining trend. At a cement replacement ratio of 50%, the compressive strength was approximately 5.6 MPa, while the tensile strength was 0.376 MPa. When the cement replacement ratio increased to 60%, the compressive strength decreased to approximately 4.4 MPa, and the tensile strength decreased to 0.349 MPa. With a further increase to 70%, the compressive strength further decreased to approximately 2.8 MPa, and the tensile strength dropped to 0.274 MPa. Compared with the 50% replacement ratio, the compressive and tensile strengths at the 70% replacement ratio decreased by approximately 50.0% and 27.1%, respectively, indicating that an excessive cement replacement ratio significantly weakens the load-bearing capacity of the backfill.

The reduction in strength with increasing cement replacement ratio can be attributed to changes in the binder composition and hydration process. When the cement replacement ratio increases, the relative cement clinker content in the binder decreases, which weakens the early hydration reaction and reduces the formation of primary hydration products such as C-S-H gel. At the same time, the increased proportion of fly ash may produce a dilution effect at early curing ages, resulting in a lower amount of effective cementitious phases and a slower development of matrix bonding. Although fly ash can contribute to strength development through secondary pozzolanic reactions, this reaction is generally slower than cement hydration and may not fully compensate for the reduction in cement content within the curing period considered in this study. As a result, the matrix becomes less compact, and the bonding capacity among coal gangue, desert sand, fly ash particles, and rice husk is weakened. Since the reinforcing contribution of rice husk depends on sufficient encapsulation and interfacial contact with the cementitious matrix, an insufficiently developed binder structure may limit its ability to contribute to crack resistance and post-peak deformation behavior. This interpretation is further discussed in the subsequent SEM analysis, where the matrix compactness, hydration products, and reinforcement–matrix interfacial features are examined from a microstructural perspective.

Curing age has a pronounced positive effect on the strength development of the RH-5% backfill. As the curing age increased from 3 to 28 days, the peak stress of the stress–strain curves continuously increased. The compressive strength increased from approximately 1.4 MPa to approximately 5.6 MPa, with the 28-day strength being about four times that at 3 days. The tensile strength increased from 0.163 MPa at 3 days to 0.331 MPa at 7 days and reached 0.376 MPa at 28 days, representing an increase of approximately 130.7% compared with the 3-day value. Notably, the tensile strength increased rapidly from 3 to 7 days, whereas the growth rate decreased from 7 to 28 days, suggesting that the RH-5% backfill exhibits pronounced early-age strength development and gradually stabilizes at later curing ages.

Overall, at a rice husk dosage of 5%, increasing the cement replacement ratio reduces the mechanical performance of the backfill, whereas extending the curing age promotes strength development. The specimen with a cement replacement ratio of 50% and a curing age of 28 days exhibits the best overall performance among the tested groups. These results indicate that the mechanical contribution of natural rice husk is closely related to the development of the cementitious matrix. A sufficiently compact matrix and adequate interfacial contact are necessary for rice husk to participate in crack resistance and deformation coordination during loading.

### 3.3. AE-DIC Damage Evolution and Crack Propagation Analysis

#### 3.3.1. AE Ringing Count Characteristics

**Figure 10:**
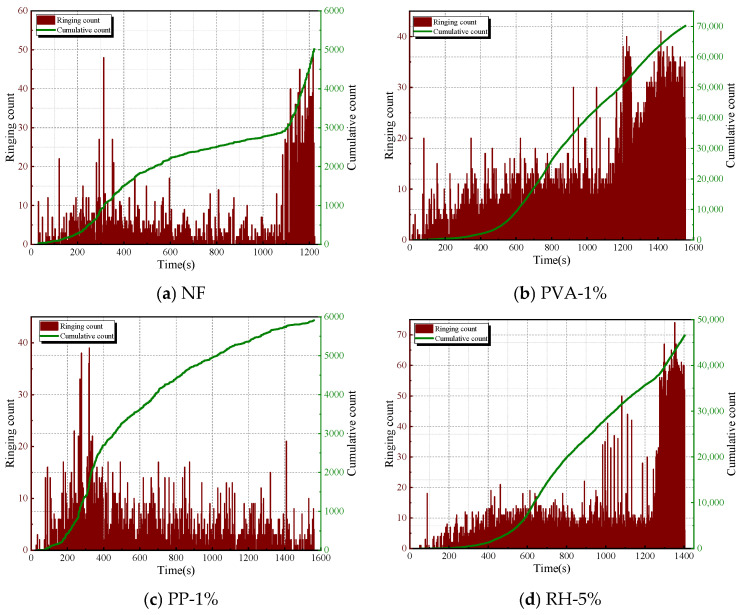
AE ringing counts of different fiber-reinforced backfills with loading time. Note: NF denotes the non-fiber group, and RH denotes natural rice husk. All groups had a cement replacement ratio of 50% and curing age of 28 d.

To characterize the intensity of damage activity and the stages of crack propagation during loading, four groups of specimens, namely H-0%, PVA-1%, PP-1%, and RH-5%, were selected for acoustic emission (AE) ringing count analysis. The results are shown in Figure 10. Since a two-sensor AE configuration was adopted in this study, the analysis mainly focuses on the evolution of ringing count and cumulative ringing count with loading time, while spatial crack localization was not performed.

As shown in Figure 10, the AE ringing count of all specimens exhibits clear staged characteristics during loading. At the initial loading stage, the ringing count remains relatively low, indicating that the internal response of the specimens is mainly associated with pore compaction and closure of pre-existing microcracks. As loading proceeds, the ringing count gradually increases, suggesting the initiation and stable development of internal damage. Near the failure stage, the ringing count increases markedly, corresponding to rapid crack propagation and coalescence.

The AE evolution characteristics differ among the tested groups. For the fiber-free group H-0%, AE activity remains relatively weak during the early and middle loading stages, whereas a concentrated and abrupt increase in ringing count occurs shortly before failure. This indicates that damage accumulation in the fiber-free specimen is limited before peak loading, followed by rapid crack coalescence and brittle failure. The PVA-1% group exhibits the highest cumulative ringing count, reflecting more sustained damage activity during loading and a more progressive failure process. The AE response of the PP-1% group is intermediate between those of the H-0% and PVA-1% groups, indicating that PP fibers also modify the damage evolution process, although their effect is weaker than that of PVA fibers.

For the RH-5% group, the cumulative ringing count is higher than that of the H-0% group, and AE activity continues to develop during the middle and late loading stages. This indicates that the incorporation of an appropriate amount of natural rice husk changes the damage evolution behavior of the backfill and delays the abrupt coalescence of cracks to some extent. Compared with the H-0% group, the RH-5% group exhibits a more progressive damage process, with less abrupt damage accumulation before final failure.

#### 3.3.2. DIC Surface Deformation and Crack Propagation Analysis

**Figure 11:**
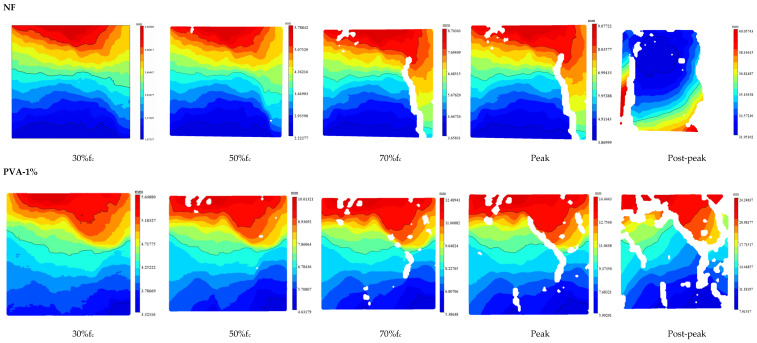

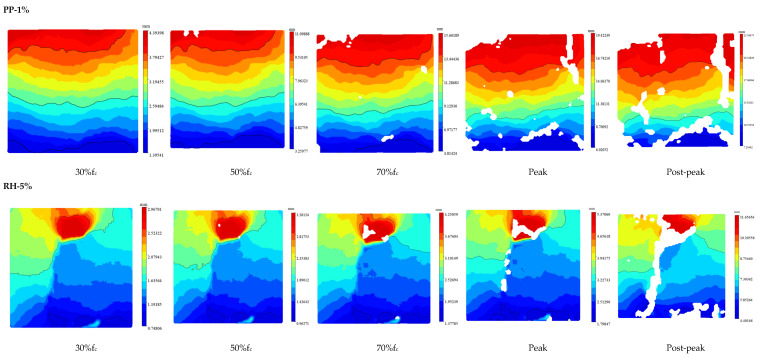
DIC surface displacement field evolution of different fiber-reinforced backfills. Note: NF denotes the non-fiber group, and RH denotes natural rice husk. All groups had a cement replacement ratio of 50% and curing age of 28 d.

To further investigate the surface deformation and crack propagation characteristics of different fiber-reinforced backfills during loading, digital image correlation (DIC) was used to obtain the surface resultant displacement contours of each group at 30%f_c_, 50%f_c_, 70%f_c_, the peak stage, and the post-peak stage, as shown in Figure 11. The color variation in the contours reflects differences in surface displacement distribution. The white decorrelation regions generally appear at locations with abrupt displacement changes and can therefore be used to assist in identifying crack propagation or localized failure zones.

As shown in Figure 11, the displacement field of the H-0% group remains relatively continuous at 30%f_c_ and 50%f_c_, indicating that the specimen is mainly in the compaction and elastic deformation stages. When the load reaches 70%f_c_ and the peak stage, evident displacement discontinuities and decorrelation regions gradually appear on the right side of the specimen. After the peak, the displacement field changes substantially, indicating rapid propagation of the dominant crack and localized failure. This reflects the pronounced brittle failure behavior of the fiber-free specimen.

For the PVA-1% and PP-1% groups, the displacement fields maintain better overall continuity during loading. Although localized decorrelation regions appear at the peak and post-peak stages, a certain degree of deformation continuity is still preserved during crack propagation. This indicates that synthetic fibers can provide bridging and tensile restraint during crack development, limiting rapid crack coalescence and transforming the failure process from the concentrated abrupt failure observed in the fiber-free group to a more progressive damage process. Among them, the PVA-1% group shows relatively better post-peak displacement-field continuity, which is consistent with its higher tensile strength and cumulative AE ringing count.

Compared with the synthetic fiber groups, the RH-5% group shows localized displacement concentration even at the early loading stage. This phenomenon may be related to the heterogeneous distribution and irregular geometry of rice husk in the matrix, which can cause local stiffness differences or weak interfacial regions. As the load increases, the displacement concentration zone gradually develops and forms a relatively distinct localized failure band at the peak and post-peak stages. However, unlike the abrupt post-peak displacement-field change observed in the H-0% group, the failure of the RH-5% group develops progressively along the localized concentration zone, without rapid coalescence of a single dominant crack. This suggests that an appropriate rice husk dosage may help delay crack localization and improve the progressive failure behavior of the backfill.

Combining the AE and DIC results, the stage with a low AE ringing count corresponds to the compaction and elastic deformation stages in the DIC results, during which the displacement field remains relatively continuous. The increase in ringing count corresponds to the development of localized displacement concentration and crack propagation, whereas the abrupt increase in ringing count corresponds to the expansion of decorrelation regions and crack coalescence. Together, these results indicate that the H-0% group is characterized by concentrated brittle failure, while the PVA-1%, PP-1%, and RH-5% groups can delay crack propagation to varying degrees. In particular, the RH-5% group exhibits a certain crack-arresting and energy-dissipation capacity.

### 3.4. SEM Micromorphology and Reinforcement Mechanism

**Figure 12:**
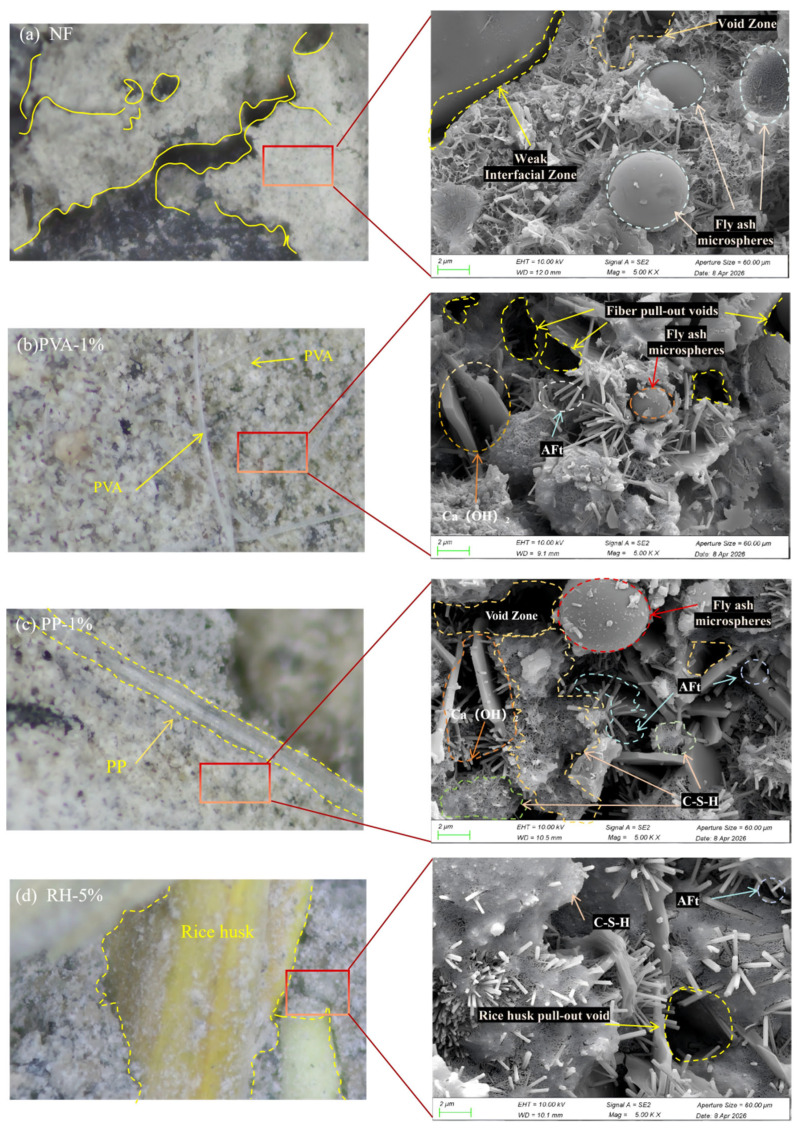
SEM micromorphologies of different fiber-reinforced backfills. Note: NF denotes the non-fiber group, and RH denotes natural rice husk. All groups had a cement replacement ratio of 50% and curing age of 28 d.

A combined fine-scale and microscopic analysis was conducted using an industrial magnifier (Sunny Optical Technology Co., Ltd., Yuyao, China) and scanning electron microscopy (SEM; ZEISS, Oberkochen, Germany). As shown in Figure 12, the macroscopic crack morphology and microstructure of the fiber-reinforced backfill specimens differ to some extent depending on the type of reinforcement.

For the fiber-free specimen (NF), surface cracks are relatively continuous and locally tend to penetrate through the specimen. The SEM images reveal numerous pores and weak interfacial regions between particles and hydration products. In particular, the contact between fly ash microspheres and hydration products is relatively loose, indicating an insufficiently compact matrix structure that can provide preferential paths for crack development. For the PVA fiber group, the macroscopic image shows fibers spanning across surface cracks. The SEM images further show fiber pull-out traces and hydration products such as AFt and Ca(OH)_2_ near the fiber surface. The fiber–matrix interfacial transition zone is partly filled with hydration products, suggesting that the PVA fibers are locally bonded with the hardened matrix. However, small interfacial gaps and local defects are still observed around some fibers, indicating that the interfacial bonding is not completely continuous. For the PP fiber group, the fibers are distributed in a relatively linear form within the matrix. C-S-H gel, AFt, and fly ash microspheres are observed around the fibers, forming a locally denser structure than that of the NF specimen. Nevertheless, local pores and interfacial gaps remain in the fiber–matrix transition zone. These observations indicate that the interaction between PP fibers and the matrix is mainly associated with physical contact and partial embedding rather than a fully bonded interface. For the RH-5% specimen, the magnified optical image shows natural rice husk embedded in the cementitious matrix, with direct contact between rice husk and the surrounding hardened backfill. The SEM image further reveals flake-like or strip-like rice husk within the matrix, together with hydration products such as C-S-H gel and AFt around the rice husk. These hydration products partially fill the adjacent pore space and improve the local compactness of the matrix. In addition, rice husk pull-out voids are observed in the SEM image, reflecting local interfacial debonding and pull-out during crack development. The rough surface and embedded morphology of rice husk are conducive to mechanical contact with the cementitious matrix and disturbance of the crack propagation path.

Overall, the NF specimen exhibits a relatively loose microstructure, with more visible pores and weak interfacial regions, which is consistent with its brittle macroscopic response. In contrast, the PVA, PP, and RH-5% specimens show denser local microstructures and clearer reinforcement–matrix interfacial characteristics. For the RH-5% specimen, the combined optical and SEM observations reveal that natural rice husk forms local contact and embedding with the cementitious matrix rather than acting only as a loose inclusion. This multiscale morphological evidence provides microstructural support for the reinforcing effect of natural rice husk in the backfill matrix.

## 4. Conclusions

(1) The type of reinforcing material strongly influenced the mechanical behavior of the backfill. Among the tested groups, PVA fibers provided the most pronounced improvement, followed by PP fibers, indicating that conventional synthetic fibers remain effective for enhancing the strength and post-peak deformation behavior of coal-based solid-waste backfill. The comparison with the fiber-free group further confirms that fiber incorporation is beneficial for reducing the brittle failure tendency of the backfill.

(2) Natural rice husk showed a clear dosage-dependent effect. A low rice husk dosage produced limited improvement, whereas a dosage of 5% provided a better balance among strength, post-peak deformation behavior, and crack resistance. However, further increasing the rice husk dosage to 10% reduced the mechanical performance, suggesting that excessive rice husk may disturb the compactness and continuity of the cementitious matrix. Therefore, the reinforcing contribution of natural rice husk depends on a suitable dosage range and adequate interaction with the matrix.

(3) The cement replacement ratio and curing age played important roles in the performance development of the RH-5% backfill. A higher cement replacement ratio weakened the mechanical performance, highlighting the importance of maintaining sufficient cementitious binder to ensure matrix integrity and effective contact between the matrix and rice husk. In contrast, prolonged curing promoted strength development, indicating that the reinforcing effect of natural rice husk is closely related to the formation and development of the cementitious matrix.

(4) The coupled AE–DIC results showed that the fiber-free specimen exhibited abrupt damage accumulation and localized brittle failure, whereas the PVA, PP, and RH-5% specimens showed more progressive damage evolution to varying degrees. For the RH-5% specimen, the continued development of AE activity and the gradual evolution of localized displacement indicate that an appropriate amount of natural rice husk can delay rapid crack coalescence and improve the progressive failure behavior of the backfill.

(5) The combined optical and SEM observations showed that natural rice husk in the RH-5% specimen formed local contact and partial embedding with the cementitious matrix. Hydration products around the rice husk, together with its rough surface, pull-out voids, and interfacial features, provide microstructural support for the interaction between natural rice husk and the hardened matrix. These observations support the feasibility of using natural rice husk as a low-cost and renewable reinforcing material in coal gangue–desert sand–fly ash-based mine backfill.

The present conclusions are limited to the material compositions, rice husk dosages, cement replacement ratios, and curing conditions investigated in this study. Further studies are needed to evaluate the long-term durability, slurry transport behavior, and field applicability of natural rice husk-reinforced backfill materials.

## Figures and Tables

**Table 1:** Oxide composition of coal gangue determined.

Oxide Composition	SiO_2_	Al_2_O_3_	Fe_2_O_3_	K_2_O	CaO	TiO_2_	SO_3_	MgO	Na_2_O	P_2_O_5_
**Mass fraction/%**	57.68	26.97	4.66	2.79	2.15	1.63	1.51	1.18	0.78	0.30

**Table 2:** Particle size distribution of coal gangue aggregates.

Particle Size Range/mm	<0.16	0.16~0.315	0.315~0.63	0.63~1.25	1.25~2.5	2.5~5	>5
**Cumulative retained/%**	0.0	15.0	33.0	50.0	80.0	98.0	100.0

**Table 3:** Chemical composition of cement determined.

Chemical Composition	CaO	SiO_2_	Al_2_O_3_	Fe_2_O_3_	SO_3_	MgO	K_2_O	Na_2_O	MnO	P_2_O_5_
**Mass fraction/%**	56.60	24.70	5.28	3.71	2.76	2.34	0.64	0.54	0.42	0.15

**Table 4:** Mineralogical composition of cement determined.

Mineral Phase	C_3_S	C_2_S	C_3_A	C_4_AF	Gypsum	Quartz	Calcium Hydroxide
**Content/%**	59.33	13.25	12.13	8.48	4.27	0.99	1.56

**Table 5:** Physical properties of fly ash.

Mean Particle Size (μm)	Water Demand Ratio (%)	Loss on Ignition (%)	SO_3_ Content (%)	28 d Strength Activity (%)
6.7	92	1.48	0.64	85

**Table 6:** Chemical composition of fly ash determined by XRF analysis.

Chemical Composition	SiO_2_	Al_2_O_3_	Fe_2_O_3_	CaO	K_2_O	TiO_2_	SO_3_	MgO	Na_2_O	P_2_O_5_
**Mass fraction/%**	49.08	36.29	5.67	2.89	1.58	1.38	1.34	0.57	0.46	0.31

**Table 7:** Mineralogical composition of fly ash determined.

Mineral Phase	Mullite	Quartz
**Content/%**	95.23	4.77

**Table 8:** Properties of natural rice husk and fibers.

Fiber Type	Fiber Length (mm)	Density (g/cm^3^)	Tensile Strength (MPa)	Elastic Modulus (GPa)	Elongation at Break (%)
Natural rice husk	5–10	0.72–1.2	15~70	3.5	1
PP	12	0.90–0.92	300–600	3.6	15
PVA	12	1.28–1.31	1830.0	40.0	7

**Table 9:** Experimental design.

Group	Cement Replacement Ratio (%)	Fiber Type	Fiber Content (%)	Curing Age
NF-C50-28d	50	NF	0	28 d
PP1-C50-28d	50	PP	1	28 d
PVA1-C50-28d	50	PVA	1	28 d
RH1-C50-28d	50	RH	1	28 d
RH5-C50-3d	50	RH	5	3 d
RH5-C50-7d	50	RH	5	7 d
RH5-C50-28d	50	RH	5	28 d
RH10-C50-28d	50	RH	10	28 d
RH5-C60-28d	60	RH	5	28 d
RH5-C70-28d	70	RH	5	28 d

## Data Availability

The original contributions presented in this study are included in the article. Further inquiries can be directed to the corresponding author.
